# Who Reduces Working Hours Before Retirement? A Longitudinal Study On Plans And Behaviors In The Netherlands

**DOI:** 10.1093/geroni/igaf122.901

**Published:** 2025-12-31

**Authors:** Camilla Marabini, Marleen Damman, Kene Henkens

**Affiliations:** Netherlands Interdisciplinary Demographic Institute, The Hague, Zuid-Holland, Netherlands; Utrecht Universiy, Utrecht, Utrecht, Netherlands; Netherlands Interdisciplinary Demographic Institute, The Hague, Zuid-Holland, Netherlands

## Abstract

As retirement ages increase, reducing working hours before retirement is seen as a way to facilitate longer working lives of those workers struggling with work demands. The current study analyzes individual, household, job, and organizational factors that shape the decision to reduce work hours before retirement. Three key questions are examined: Who plans to reduce working hours before retirement, who is actually reducing working hours, and what explains the discrepancy between the two? Using two waves of the NIDI pension panel survey, we examine the plans to reduce work hours of 4243 employees aged 60-63 nested in 1086 organizations, and track their actual behavior after retirement. Results suggest that around 20% of the sample plans to reduce working hours in the years before retirement age. Older workers with poor health, and higher income were more likely to plan to reduce working hours. In the follow-up years, 30% of the older workers actually reduced working hours. Plans proved to be strong predictors of actual behavior. Within those who plan to reduce working hours before retirement, around half actually reduces them; around 30% of those who were uncertain ends up reducing work hours; and a fifth of those who planned to work full-time until retirement eventually reduces work hours. Findings from this study contribute to scientific debates about agency during later working lives and to policy debates about gradual retirement pathways.

